# Bcl-B: an “unknown” protein of the Bcl-2 family

**DOI:** 10.1186/s13062-023-00431-4

**Published:** 2023-10-30

**Authors:** N. V. Pervushin, G. S. Kopeina, B. Zhivotovsky

**Affiliations:** 1 Engelhardt Institute of Molecular Biology, Russian Academy of Sciences, Moscow, 119991 Russia; 2 Faculty of Medicine, MV Lomonosov Moscow State University, Moscow, 119991 Russia; 3 Division of Toxicology, Institute of Environmental Medicine, Karolinska Institute, Box 210, Stockholm, 17177 Sweden

**Keywords:** Bcl-B/Bcl-2L10, Bcl-2 family proteins, Carcinogenesis, Programmed cell death, Oogenesis, Embryogenesis

## Abstract

Bcl-B is a poorly understood protein of the Bcl-2 family that is highly expressed in many healthy tissues and tumor types. Bcl-B is considered an antiapoptotic protein, but many reports have revealed its contradictory roles in different cancer types. In this mini-review, we elucidate the functions of Bcl-B in normal conditions and various pathologies, its regulation of programmed cell death, its oncogene/oncosuppressor activity in tumorigenesis, its impact on drug-acquired resistance, and possible approaches to inhibit Bcl-B.

## Introduction

The members of the Bcl-2 family of proteins are essential regulators of cell death which control activation of the intrinsic pathway of apoptosis. They are divided into two groups: pro- and antiapoptotic proteins. The former is also separated into two subsets: multidomain effector proteins (Bak and Bax) and regulatory proteins containing only one Bcl-2 homology (BH) region, namely BH3-only proteins (Bim, Bid, Bad, Bmf, Bik, Noxa, Puma, and Hrk). The induction of the intrinsic pathway in response to various stimuli leads to displacement of proapoptotic proteins from their antiapoptotic partners, mitochondrial outer membrane permeabilization, and activation of the caspase cascade that ultimately results in apoptotic cell death. The increased level of prosurvival proteins is responsible for evasion of cell death and promotes carcinogenesis [1–3].

**Fig. 1:**
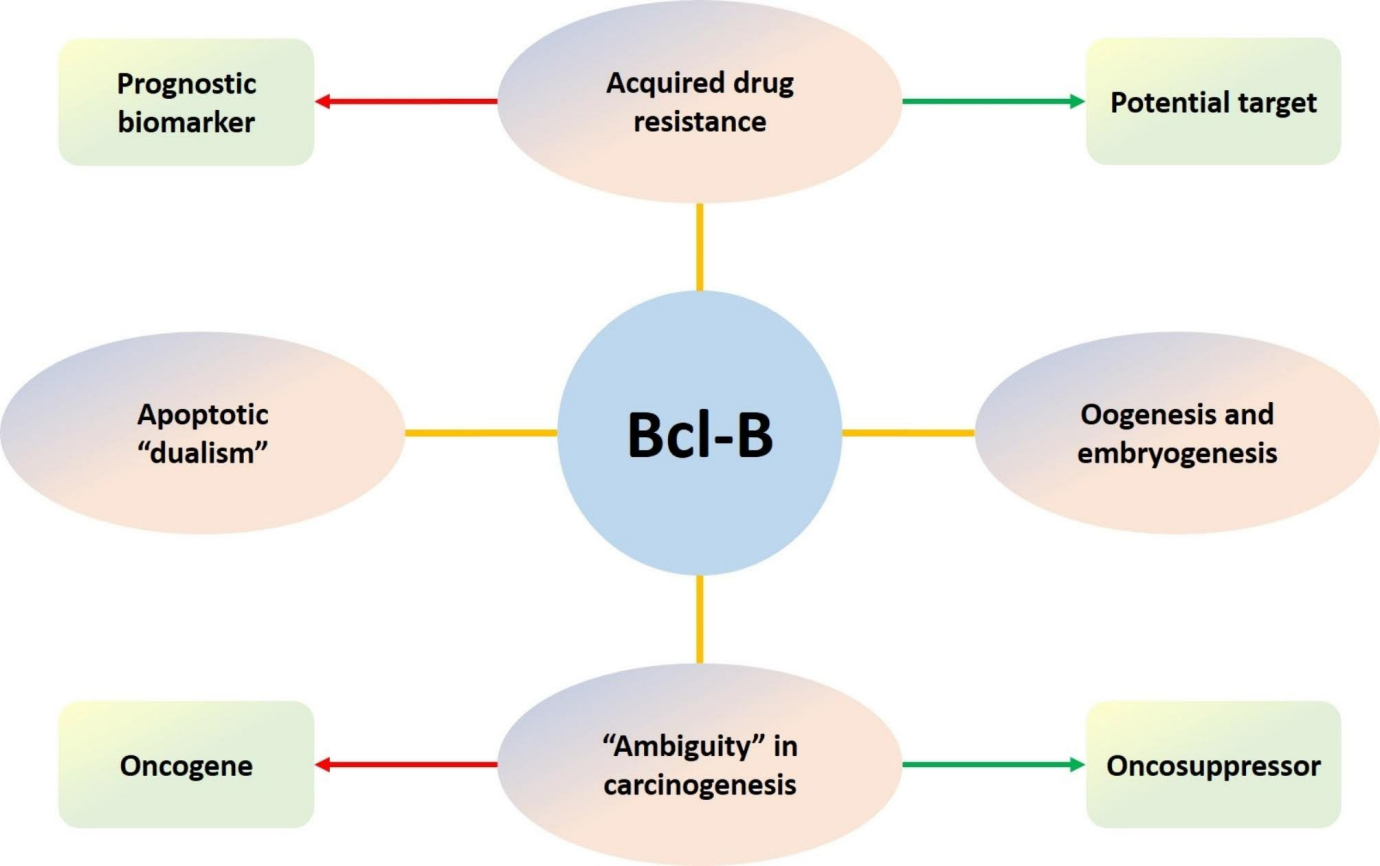
The roles of Bcl-B in normal and pathological conditions

The antiapoptotic subfamily includes at least six members (Bcl-2, Bcl-xL, Mcl-1, Bcl-w, Bfl-1, and Bcl-B) [3]. The first three proteins have been studied intensively by many authors [4–6]. Recently, Bcl-w and Bfl-1 have also been discussed in detail [7, 8]. However, despite its apoptotic and non-apoptotic functions, Bcl-B remains poorly characterized. In this mini-review, we try to close this gap and summarize the current knowledge concerning the functions of this “unknown” protein (Fig. 1).

## Discovery, structure, and protein–protein interactions

Bcl-B (B-cell lymphoma 2 family protein resembling Boo)/Bcl-2L10/Nrh (Bcl-2 like protein 10), is encoded by *BCLB* gene. This human protein was discovered independently by three groups in 2001 [9–11]. Bcl-B is homologous to the murine Bcl-2 protein Boo/Diva, a fact that is reflected in its name [9]. Despite the structural resemblance (the amino acid sequence identity and similarity between Bcl-B and Boo is about 45.5–47% and 60.7%, respectively), there are some differences between these proteins [9, 12]. First, Bcl-B contains 204 amino acids, while Boo is only 191 amino acids long protein [13]. Second, human Bcl-B is widely expressed in many healthy tissues and tumors in adults, while Boo has been found mainly in mouse ovary and testis [9, 14].

Surprisingly, more than twenty years after its discovery, many aspects of Bcl-B remain unclear. The first “open question” is the structural organization of Bcl-B. On the one hand, numerous researchers have reported that Bcl-B has a structure that is typical for various antiapoptotic proteins of the Bcl-2 family [15], with four BH domains (BH1, BH2, BH3-like, and BH4) and a transmembrane (TM) domain [9, 16–20]. On the other hand, some researchers have presented evidence that human Bcl-B/Bcl-2L10 and murine Boo/Diva are characterized by the absence of a BH3 domain [10, 12, 13, 21]. Moreover, Boo/Diva contains an altered BH1 domain that prevents possible interactions with proapoptotic proteins [13].

The second “open question” relates to possible interaction partners of Bcl-B. This protein is able to interact with fewer Bcl-2 family proteins compared with the other antiapoptotic Bcl-2 family proteins. Bcl-B/Bcl-2L10 selectively binds Bax and neutralizes its proapoptotic function [9, 17, 18, 22]. Mutant forms of any of these proteins abrogate the formation of the corresponding complex [18]. However, Bcl-B cannot interact with Bak, Bad, and Bid [18]. Surprisingly, Boo/Diva can bind Bak, but not Bax [21]. Another partner of Bcl-B is the BH3-only protein Bim. Interestingly, Bim and Bax form complexes with the whole subset of the antiapoptotic Bcl-2 family proteins [12]. The main reason for specificity of Bcl-B binding to only these two proteins is still unclear.

Finally, it should be noted that Boo/Diva knockout mice show no abnormalities. The mice are fertile, and their life expectancy is similar to that of wild type mice [23]. The lack of abnormalities suggests several things. First, the functional activity of Bcl-B during embryogenesis could be compensated by other proteins. Second, Bcl-B targeting in humans may result in lower negative effects in normal tissues compared with other proteins of the Bcl-2 family. Notably, Bcl-xL or Mcl-1 knockout in mice leads to embryonic lethality [24, 25]. Moreover, selective inhibitors of Bcl-xL and Mcl-1 have not succeeded in clinical trials due to excessive toxicity [2, 26]. Bcl-2 knockout in mice results in an altered phenotype. Venetoclax, a selective Bcl-2 inhibitor, was approved by the Food and Drug Administration (FDA) several years ago for treatment of various cancers [2].

### The role of Bcl-B in normal conditions

#### Pro- and antiapoptotic properties

Another unclear area is the functional activity of Bcl-B. Is it an apoptotic or a prosurvival protein? Again, the results are controversial. Murine Boo/Diva has demonstrated both proapoptotic [20, 27] and antiapoptotic [21, 28, 29] activities in different in vitro cell models. For example, nucleoside diphosphate kinase NM23-H2 suppresses Bcl-B- or Boo-mediated apoptosis in vitro [16]. According to various reports, human Bcl-B/Bcl-2L10 exerts predominantly antiapoptotic properties [9, 10, 17, 18]. However, Nur77/TR3, an orphan nuclear receptor, can bind to Bcl-B and transform its antiapoptotic phenotype into a proapoptotic one via conformational changes in its structure that expose its BH3 domain and subsequent exertion of proapoptotic activity in plasma and myeloma cells [30, 31]. Of note, Nur77/TR3-dependent transformation has been proposed for Bcl-2 [32]. Importantly, Nur77/TR3 does not contain BH domains. This fact suggests that Bcl-B could interact with other proteins not just via the BH-mediated interface. It is likely that the pro- or antiapoptotic role of Bcl-B and Diva is determined by the cellular context, but this topic requires further investigation.

#### Autophagy

A possible explanation for the contradictory apoptotic functions of Bcl-B could be that this protein regulates other types of programmed cell death (PCD), in particular, autophagy, which promotes degradation of damaged proteins and organelles. This process might also act as an essential adaptive mechanism for the maintenance of cell survival by preventing apoptosis [33, 34]. Bcl-B binds to the BH3 domain of Beclin 1 and can block this important activator of autophagy. In contrast, Bcl-B suppression induces both apoptosis and autophagy [19, 35]. A similar mechanism of autophagy regulation is known for Bcl-2 and Bcl-xL [36, 37].

Mitophagy is a subtype of autophagy and represents selective elimination of aged and damaged mitochondria in lysosomes. Mitophagy activation usually inhibits apoptosis, but it is also able to promote apoptosis in several situations. Bcl-B/Bcl-2L10 can control the activity of Parkin, an E3 ubiquitin ligase and a key participant of mitophagy. The formation of a Bcl-B/phospho-Parkin complex blocks mitophagy and thus inhibits apoptosis in hepatic stellate cells [38]. Taken together, the disturbed balance between apoptosis and autophagy regulation can underlie the “apoptotic dualism” of Bcl-B (Fig. 1).

The functional activity of all proteins is linked to their subcellular localization. Like other antiapoptotic proteins of the Bcl-2 family, Bcl-B contains a C-terminal TM domain that is responsible for its anchoring in the intracellular membranes. Bcl-B is commonly located in the outer mitochondrial membrane (OMM) [15]. However, prosurvival proteins can also be localized in the endoplasmic reticulum (ER) to regulate intracellular Ca^2+^ levels and activation of apoptosis [39]. Bcl-B can bind to the inositol 1,4,5-trisphosphate receptor (IP3R) through its BH4-domain and block Ca^2+^ release from the ER, thereby preventing apoptosis. Bcl-B-mediated regulation of Ca^2+^ is controlled by IP3R-binding protein (IRBIT): Phospho-IRBIT enhances the action of Bcl-B, but dephosphorylation of IRBIT has the opposite effect [40]. Taken together, these data contribute “contradictions” regarding the apoptotic properties of Bcl-B.

#### Oogenesis and embryogenesis

Bcl-B and its homologs are highly expressed in mice, buffalo, zebrafish, and human oocytes; ovarian tissue; and early-stage embryos. This protein plays an important role in the development and maintenance of oocytes and embryos [41–46]. Boo/Diva and BCL2L10 suppression inhibits oocyte maturation in cultured murine and buffalo oocytes [42, 45], a phenomenon accompanied by alterations in their spindles and chromosome organization [42]. Moreover, Bcl-B is essential for correct microtubule organization in mouse and human oocytes [43, 47]. Interestingly, Bcl-B is mainly found in the cytosol of human oocytes and embryos, whereas in adult tissues it is localized in mitochondria and the ER. Meanwhile, Bcl-B is detected in the nucleus of abnormal embryos and might be a potential biomarker of “embryo quality” [43, 44]. The zebrafish protein Nrz (a homolog of murine Boo) located in the OMM and ER and regulates apoptosis and Ca^2+^ signaling, thereby controlling cytoskeletal dynamics. It is essential for processes of gastrulation and somitogenesis in zebrafish [39, 48]. Additionally, blastocysts of patients with polycystic ovaries have lower expression of *BCL2L10* compared with healthy controls [49]. Taken together, these observations indicate that Bcl-B is crucial for the maintenance of oogenesis and embryogenesis in humans and animals, underlining the importance of apoptosis regulation in physiological processes.

### The regulation of Bcl-B and its role in pathology

#### Transcriptional/translational level

**Fig. 2:**
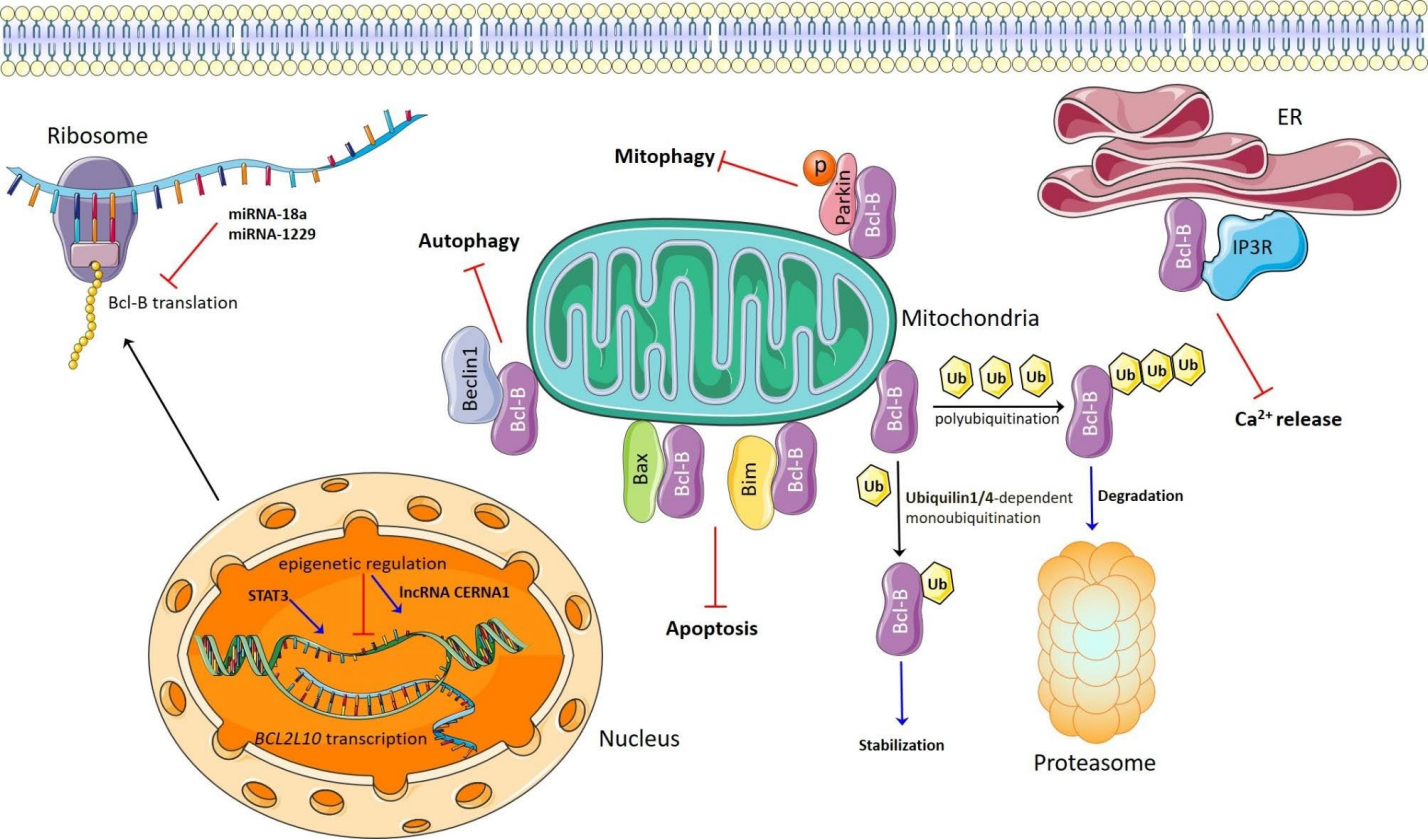
The regulation of Bcl-B and its participation in programmed cell death. P – phosphorylated form of the Parkin protein; Ub – ubiquitin. The figure was prepared by using the elements from Servier Medical Art, which is licensed under a Creative Commons Attribution 3.0 Unported License

Like all proteins, Bcl-B is regulated at the transcriptional, translational, and posttranslational levels (Fig. 2).

Unfortunately, transcriptional and translational regulation of Bcl-B is understudied. STAT3 is a positive regulator of Bcl-B transcription in melanoma [50]. Additionally, the long noncoding RNA *CERNA1* increases the Bcl-2L10 transcription rate via epigenetic regulation in vascular endothelial cells and ovarian cancer [51, 52]. At the translational level, several microRNAs (miRNAs) have been reported to regulate Bcl-B/Bcl-2L10 expression. First, miRNA-1229 negatively affects the Bcl-2L10 level in colorectal cancer [53]. miRNA-18a also downregulates Bcl-2L10, and this change might mediate cell invasion, migration, and proliferation in hepatocellular carcinoma (HCC) [54]. Moreover, miRNA-dependent regulation of Bcl-B might contribute to the development of non-cancer diseases. For example, toxic epidermal necrolysis (a type of severe drug-induced skin reaction) whose precise pathogenesis remains unknown could be associated with excessive keratinocyte apoptosis. Moreover, miR-18a-5p-mediated Bcl-B suppression activates apoptotic death of keratinocytes in patients with toxic epidermal necrolysis [55, 56].

#### Posttranslational level

The process of proteasomal degradation is an important mechanism by which Bcl-B is regulated [57]. Ubiquilin-1 is a selective Bcl-B regulator that does not interact with other antiapoptotic Bcl-2 family proteins. Ubiquilin-1-dependent monoubiquitinylation of Bcl-B leads to its stabilization and removal from mitochondria to the cytosol [58]. Furthermore, another member of the ubiquilin family, ubiquilin-4, stabilizes Bcl-B and prevents apoptosis in mesothelioma cells, which have high Bcl-B expression [59]. Unfortunately, the mechanisms underlying the stabilization and relocation of Bcl-B after interactions with ubiquilins remain uncertain and require further clarification.

It should be noted that stability of antiapoptotic proteins correlates with their prosurvival activity. Of the six antiapoptotic Bcl-2 family proteins, Bcl-B, Bfl-1, and Mcl-1 are more prone to basal or drug-mediated proteasomal turnover in cancer cells; this turnover could limit their functional activity. Nevertheless, disturbances in the proteasomal degradation machinery of Bcl-B, Bfl-1, and Mcl-1 could facilitate drug resistance or tumor development [60]. Notably, the indirect inhibitor (PaTrin-2) of the specific deubiquitinase of Mcl-1 has been studied [1]. Furthermore, the combination of azacytidine (a chemotherapeutic agent) and erlotinib (an inhibitor of the epidermal growth factor receptor) has a synergetic effect in acute myeloid leukemia (AML) cells and primary AML, and myelodysplastic syndrome (MDS) cells by inducing proteasomal degradation of Mcl-1 and Bcl-B; these data indicate the indirect inhibition of Bcl-B [61]. These facts suggest that the cellular strategies used to control the levels of antiapoptotic Bcl-2 family proteins can be applied for therapy. Accordingly, targeting deubiquitinases or activating ubiquitin ligases of Bcl-B appears to be a potential strategy to eliminate Bcl-B-dependent cancer cells (Fig. 2).

#### “Dualism” of Bcl-B in carcinogenesis: Oncogene/oncosuppressor activity

As mentioned earlier, Bcl-B is highly expressed in normal and tumor tissues. Its expression has been detected in many types of solid and blood malignancies [14]. However, its role in tumor development and progression is variable. Bcl-B acts as an oncogene in some tumors [50, 62–64] but prevents tumorigenesis in others [65–67]. Hence, the following question arises: what are the reasons underlying the “ambiguity” of Bcl-B in carcinogenesis?

It is well known that evasion of cell death is one of the hallmarks of cancer that can be achieved by increased expression of the antiapoptotic Bcl-2 family proteins [1, 68]. Indeed, upregulated Bcl-B levels are responsible for tumor promotion in cases of breast cancer [62], melanoma [50, 63], and multiple melanoma (MM) [69]. At the same time, the Bcl-B level correlates with a positive prognosis in patients with HCC and gastric cancer [70, 71]. Moreover, this protein blocks cell migration, angiogenesis, and metastasis, thereby, serving as a oncosuppressor in HCC [70]. How might this be possible?

First, it could be associated with the “apoptotic dualism” of Bcl-B (Fig. 1). Besides its prosurvival activity, Bcl-B is also involved in the regulation of autophagy and Ca^2+^ signaling, as discussed above. Indeed, Bcl-B can stimulate autophagy in HCC [66], an action that could explain its tumor suppressor activity in this cancer type. Second, the potential mutations in the protein structure of Bcl-B or *BCL2L10* polymorphisms could abate its antiapoptotic activity. For example, a *BCL2L10* single nucleotide polymorphism (rs2231292, Leu11Arg), which is predicted to be a biomarker of a favorable outcome, has been observed in patients with breast and rectal cancer. It leads to disturbance of the interactions between BCL2L10 and IP3R that, in turn, facilitates Ca^2+^ release from the ER and activation of Ca^2+^-dependent cell death [72, 73]. Additionally, patients with this BCL2L10 polymorphism have a diminished risk of the development *de novo* MDS [74]. Third, epigenetic regulation of Bcl-B has great significance in tumorigenesis: Methylation of the gene could lead to silenced or reduced expression of this protein in HCC [66] and gastric cancer [65, 75, 76]. Finally, a decrease in level of one antiapoptotic protein can be compensated for by an increase in the level of another prosurvival partner, a phenomenon that has been proved in various in vitro and in vivo models. Therefore, mutant cells might contain “low” levels of Bcl-B and “high” levels of Bcl-2, Bcl-xL, Mcl-1, etc. Indeed, the survival of various cancer cells is “dependent” on distinct antiapoptotic proteins [1, 2, 77, 78].

## Conclusions

Bcl-B/Bcl-2L10 is a multifaceted protein that exerts both pro- and antiapoptotic activities, allowing it to act as an oncogene as well as an oncosuppressor in different cancer types. These “dual” activities are possibly associated with its negative epigenetic regulation, altered structure due to gene polymorphism, and regulation of other types of PCD. Some data suggest that Bcl-B is involved in autophagy [19, 35] and Ca^2+^-mediated apoptosis [39, 40]. This protein can probably control other types of PCD, an eventuality that should be elucidated in the near future. Bcl-B participates in oogenesis and embryogenesis, but its knockout in mice does not lead to any negative effects [23], a finding that could be promising in the context of Bcl-B targeting. At present, there is little data about Bcl-B inhibitors. This protein is prone to proteasomal degradation and could be suppressed indirectly [60, 61]. Several compounds have been reported to directly suppress Bcl-B by disrupting complexes with proapoptotic Bcl-2 family proteins *in silico* and in vitro [79–82]. Gambogic acid and gossypol, non-selective inhibitors of antiapoptotic Bcl-2 family proteins, can bind to Bcl-B [83, 84]. Importantly, high Bcl-B levels contribute to the development of acquired resistance to various chemotherapeutics, including camptotecin (a topoisomerase inhibitor) [85], busulfan (an alkylating agent) [86], ABT-737 (a non-selective BH3 mimetic) [50, 87, 88], azacytidine (a hypomethylating agent) [64, 89], and cisplatin and dacarbazine (alkylating agents) [50]. Therefore, Bcl-B could be considered an important prognostic marker in cancer. Moreover, potential blockade of Bcl-B in combination with chemotherapeutics or targeted therapy could be a promising anticancer strategy that prevents the appearance of acquired drug resistance and diminishes the possible toxic effects. Additionally, increased Bcl-B gene expression is related to some non-cancer diseases such as toxic epidermal necrolysis [55, 56], affective psychosis [90], and cardiac disorders [91]. To conclude, there is no doubt that Bcl-B plays a role in normal physiological conditions and pathologies. Further investigation will be able to resolve the current contradictions and make Bcl-B a more “understandable” protein.

## Data Availability

Not applicable.
